# Enhanced Modulation of CaMKII in Mouse Hippocampus by an Antidepressant-like Dose of Melatonin/Ketamine Combination

**DOI:** 10.3390/cells14151187

**Published:** 2025-08-01

**Authors:** Armida Miranda-Riestra, Rosa Estrada-Reyes, Luis A. Constantino-Jonapa, Jesús Argueta, Julián Oikawa-Sala, Miguel A. Reséndiz-Gachús, Daniel Albarrán-Gaona, Gloria Benítez-King

**Affiliations:** 1 Laboratorio de Neurofarmacología, Subdirección de Investigaciones Clínicas, Instituto Nacional de Psiquiatría Ramón de la Fuente Muñiz, Mexico City 14370, Mexico; armida.miranda.riestra@gmail.com (A.M.-R.); biologia0712@gmail.com (L.A.C.-J.); oikawasala@inprf.gob.mx (J.O.-S.); miguelgachus@ciencias.unam.mx (M.A.R.-G.); daniel_gaona-@hotmail.com (D.A.-G.); 2 Laboratorio de Fitofarmacología, Dirección de Investigaciones en Neurociencias, Instituto Nacional de Psiquiatría Ramón de la Fuente Muñiz, Mexico City 14370, Mexico; restrada@inprf.gob.mx

**Keywords:** melatonin, CaMKII, mayor depression, ketamine, melatonin receptors

## Abstract

Forty per cent of major depression patients are resistant to antidepressant medication. Thus, it is necessary to search for alternative treatments. Melatonin (*N*-acetyl-5-hydroxytryptamine) enhances neurogenesis and neuronal survival in the adult mouse hippocampal dentate gyrus. Additionally, melatonin stimulates the activity of Ca^2+^/Calmodulin-dependent Kinase II (CaMKII), promoting dendrite formation and neurogenic processes in human olfactory neuronal precursors and rat organotypic cultures. Similarly, ketamine, an *N*-methyl-D-aspartate receptor (NMDAR) antagonist, modulates CaMKII activity. Importantly, co-treatment of low doses of ketamine (10^−7^ M) in combination with melatonin (10^−7^ M) produces additive effects on neurogenic responses in olfactory neuronal precursors. Importantly, enhanced neurogenic responses are produced by conventional antidepressants like ISSRs. The goal of this study was to investigate whether hippocampal CaMKII participates in the signaling pathway elicited by combining doses of melatonin with ketamine acutely administered to mice, 30 min before being subjected to the forced swimming test. The results showed that melatonin, in conjunction with ketamine, significantly enhances CaMKII activation and changes its subcellular distribution in the dentate gyrus of the hippocampus. Remarkably, melatonin causes nuclear translocation of the active form of CaMKII. Luzindole, a non-selective MT_1_ and MT_2_ receptor antagonist, abolished these effects, suggesting that CaMKII is downstream of the melatonin receptor pathway that causes the antidepressant-like effects. These findings provide molecular insights into the combined effects of melatonin and ketamine on neuronal plasticity-related signaling pathways and pave the way for combating depression using combination therapy.

## 1. Introduction

Major depression affects millions of people worldwide, causing significant personal, familiar, and social burdens. Despite available treatments, around 40% of patients do not respond to treatment, and the therapeutic responses take weeks to be established, highlighting an urgent need for novel therapies [1].

Melatonin (*N*-acetyl-5-hydroxytryptamine) is an indolamine primarily synthesized in the pineal gland, and it has demonstrated antidepressant-like effects in preclinical models and humans [2,3]. However, clinical evidence about the efficacy of melatonin in humans as an antidepressant is scarce, and its effectiveness has primarily been demonstrated when it is administered in combination with another antidepressant. Only agomelatine, which acts as an agonist at melatonin MT_1_ and MT_2_ receptors and as an antagonist at serotonin 5HT_2C_ receptors, has proven efficacy and safety in humans to treat both major depression and generalized anxiety disorder [4].

Melatonin is a molecule of low molecular weight and amphiphilic in nature, enabling its diffusion across plasma membranes and distribution within subcellular compartments [5,6]. This indolamine has pleiotropic actions, and acts as a paracoid, an autocoid, and as a vitamin. It is present in vegetables, fruits, and rice, and is consumed as food by many organisms, including humans. Recently, melatonin has been compared to vitamin D due to their shared characteristics. Both function as hormones and play roles in modulating the immune, cardiovascular, and gastrointestinal systems. Additionally, they have notable effects on the skin, among other functions [7,8,9,10,11,12].

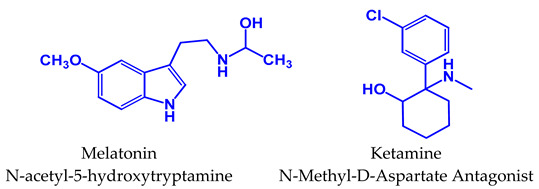



In the brain, melatonin functions as a neurotrophic factor, stimulating neurodevelopment and modulating the synthesis and release of neurotransmitters [13,14,15], and its effects are mainly mediated through MT_1_ and MT_2_ receptors [16], which activate signaling pathways promoting neuronal survival, neurogenesis, and dendritic complexity [17,18].

Importantly, melatonin has a dual effect on calcium/calmodulin-dependent protein kinase II (CaMKII) activity, stimulating or inhibiting its activity depending on experimental conditions and the microenvironment [19,20].

Inhibitory effects of melatonin on CaMKII have been related to long-term potentiation (LTP) [21], while stimulation of this enzyme by melatonin has been associated with increased dendrite formation and neurogenesis [22,23]. Stimulation and Inhibition of CaMKII activity play an important role in memory and cognition, which are cognitive functions that are diminished in major depression [24].

Ketamine, an *N*-methyl-D-aspartate receptor (NMDAR) antagonist, has gained attention for its rapid and sustained antidepressant effects, even in the treatment of resistant depression (TRD) patients [25]. At non-anesthetic doses, ketamine not only alleviates depressive symptoms but also reverses neuroplasticity deficits by promoting synaptogenesis and enhancing structural plasticity [26,27]. However, its therapeutic potential is limited by adverse effects, including psychotomimetic reactions and abuse liability, making the exploration of adjunctive strategies necessary to optimize its clinical utility [1,28].

Remarkably, CaMKII can be activated by calcium entry through NMDA receptors [29]. Calcium binds to calmodulin, inducing a conformational change. The Ca^2+^-Calmodulin complex binds to CaMKII, activating this protein [22,29].

Additionally, CaMKII participates in the signaling pathway by which ketamine mediates its antidepressant-like effects in mice. After systemic administration of TatCN21, a membrane-permeable inhibitor of CaMKII, the elicited ketamine antidepressant-like effect is abolished [30]. Importantly, CaMKII activation in hippocampal neurons triggers its own translocation and mobilization of other proteins between different cellular compartments, promoting subcellular redistribution. In this regard, the CaMKII inhibitor tatCN21 [30] blocks the redistribution of CaMKII elicited by ketamine, suggesting that CaMKII activation precludes its subcellular redistribution [31].

Our recent studies showed that combining ketamine (1.5 mg/kg) with melatonin (16 mg/kg) causes additive antidepressant-like effects in mice without affecting the ambulatory activity [32]. Furthermore, this ketamine/melatonin (KET/MEL) combination stimulates neurogenesis in human olfactory neuronal precursors [23].

CaMKII is a holoenzyme constituted by 12 subunits of 56–60 kDa assembled into two hexamers [33]. Each CaMKII subunit has three domains: the regulatory, the association, and the catalytic domain. The regulatory domain has three critical autophosphorylation sites. Among these sites, threonine 286 (T286) is susceptible to autophosphorylation by neighboring CaMKII subunits after Ca^2+^–CaM binding to the adjacent regulatory domain (For a review, see [24]). CaMKII autophosphorylation at T286 also increases the affinity of CaMKII for Ca^2+^–CaM between 10-fold and 1000-fold, making the activity of CaMKII independent of Ca^2+^, Calmodulin binding, and activation [34].

In addition, autophosphorylation at T286 in CaMKII prevents the reversion of the enzyme to an inactive conformation, even after decreases in intracellular calcium levels [35,36].

The alpha isoform of CaMKII, (*α*-CaMKII) has a molecular weight of 50 kDa. It is highly abundant in the brain. Notably, alpha-Ca^2+^–calmodulin-dependent kinase II plays a critical role in facilitating LTP within the CA1 region of the hippocampus [37].

Ketamine and melatonin have both been shown to modulate CaMKII activity, suggesting a common signaling pathway. Specifically, ketamine increases CaMKII phosphorylation in the hippocampus and the prefrontal cortex, indicating that this enzyme is in its active form [38]. Similarly, melatonin activates CaMKII in the hippocampus, promoting dendritic complexity and enhancing neurogenesis [17,22].

Given the key role of CaMKII in neurogenesis, neuronal connectivity, and its involvement in the antidepressant actions of melatonin, we assessed the relative levels of hippocampal CaMKII, phosphorylated at T286 as an index of activated CaMKII. Additionally, we examined the subcellular distribution and activity of CaMKII after acute administration of the KET/MEL combination at a dose known to induce antidepressant-like effects in mice. The role of melatonin receptors was further explored using luzindole, a non-selective melatonin receptor antagonist. Our results showed that acute administration of melatonin and the KET/MEL combination activates CaMKII downstream of melatonin receptors.

## 2. Materials and Methods

### 2.1. Animals and Pharmacological Treatments

Male Swiss Webster mice were provided by the vivarium of the Instituto Nacional de Psiquiatría Ramón de la Fuente Muñiz. Animal care and use procedures complied with the Mexican Official Norm (NOM-062-ZOO-1999) and the universal principles of laboratory animal care (NIH publication # 85-23, 2011). This study was approved by the ethics committee of the Instituto Nacional de Psiquiatría Ramón de la Fuente Muñiz (IC 22173.0).

Mice were housed in groups of 8 per cage with a controlled environment under inverted light-dark conditions (12:12 h, lights on at 22:00 h). Animals were housed in polycarbonate cages (37 × 25 × 15 cm), with controlled humidity (40–67%) and temperature (20–21 °C). Melatonin was dissolved in 0.06% ethanol saline solution, and ketamine was dissolved in saline solution. All drugs were administered intraperitoneally (ip) in a volume of 10 mL/kg body weight at the middle of the dark cycle (13:30 h).

We used four pharmacological treatments: (1) the vehicle (VEH, 0.9% NaCl saline solution + 0.06% ethanol), (2) melatonin (16 mg/kg), (3) ketamine (1.5 mg/kg), and (4) the combination of ketamine and melatonin (KET/MEL 1.5 and 16 mg/kg, respectively) and animals were subjected to FST protocol as described [20].

To measure whether binding melatonin to their receptors activates the CaMKII signaling pathway, in an independent experiment, mouse groups were treated with luzindole (Sigma-Aldrich CAS:1179 46-9-5. 3050 Spruce Street, Saint Louis, MO, USA). A total of 10 mg of luzindole was first dissolved in 100 µL of ethanol, followed by 100 µL of Tween 80 and 0.9% NaCl (saline) until 10 mL of solution was obtained. This final preparation of luzindole was used for ip administration at a 10 mg/kg body weight dose. Fifteen minutes later, a group of 5 mice per treatment were administered with either vehicle, melatonin, ketamine, or KET/MEL, at the doses described above, and 30 min after they were subjected to the FST.

### 2.2. Behavioral Assessment: Forced Swim Test

Behavioral tests were performed according to the acute administration protocol described by Estrada-Reyes et al. [3,20,32]. For the Forced Swim Test (FST), we placed each mouse individually into glass cylinders (21 cm tall and 14.5 cm in diameter) filled with water maintained at 23 ± 1 °C to a depth of 15 cm. The first swimming session of 15 min (pre-test) at 14:00 h (ZT 18). Twenty-three and a half hours later, the mice were injected with one of the pharmacological treatments: vehicle, melatonin, ketamine, or KET/MEL combination; thirty minutes later, the mice were subjected to a second swimming session of 5 min, at the end of which, they were gently dried and taken back to the house box. The immobility time of mice was recorded as described [20].

### 2.3. Sample Obtention for 2D Gel Electrophoresis Separation

For hippocampal protein separation in 2D, we used 5 mice/group of treatment. Brain tissue samples were collected immediately after the FST. Mice were euthanized by decapitation, and their brains were immediately immersed in phosphate-buffered saline (PBS 1×) at 4 °C. The hippocampi were dissected according to Madison & Edson (1997) [39] and preserved at 4 °C in lysis and extraction buffer with protease and phosphate inhibitors (RIPA Buffer by Thermo Scientific, Waltham, MA, USA). The hippocampal tissue was homogenized for 10 s with pulses of 30 Hz in an ice bath using an Ultrasonic Homogenizer from Cole Palmer (Chicago, IL, USA) three times and centrifuged at 9660× *g* at 4 °C for three minutes. Supernatants were collected and stored at −80 °C until further processing.

Samples were thawed and treated using methanol, chloroform, and distilled water, according to the manufacturer’s instructions. After centrifugation, the pellet was reconstituted in a buffer containing 8 mM urea; 2% *w*/*v* CHAPS (3-[(3-Cholamidopropyl) dimethylammonio]-1-propanesulfonate); 45 mM DTT; 2% *w*/*v* ampholytes pH 3–10, and 0.001% *w*/*v* bromophenol blue. Protein concentration was determined using the 2D Quant kit (GE Healthcare, Waukesha, WI, USA) according to the manufacturer’s instructions. This kit employs a two-phase procedure: First, quantitative precipitation of proteins via a proprietary combination of a precipitant and co-precipitant that excludes interfering substances such as EDTA from the protein pellet. Second: Solubilization of the protein pellet in a copper-containing alkaline solution, followed by a colorimetric reaction with unbound copper ions.

### 2.4. Protein Separation by Two-Dimensional Electrophoresis (2-D EF)

The hippocampal proteins (50 µg) were separated in the first dimension by isoelectric point using precast immobilized pH 3–10 gradient strips by duplicate [40]. The strips, with the proteins separated in the first dimension, were loaded onto 10% polyacrylamide gels for second-dimensional separation under denaturing conditions in the presence of Sodium dodecyl sulfate and 2-mercaptoethanol [41]. Proteins were stained with SYPRO Ruby according to the manufacturer’s protocol. Hippocampal proteins of each mouse were separated in 2D in duplicate.

### 2.5. Analysis of Proteins Separated by Two-Dimensional Electrophoresis

Gel images were obtained using the Bio-Rad Chemidoc^®^ MP Imaging System and processed with the Image Lab software v6.0 (Bio-Rad Laboratory, Hercules, CA, USA). The area under the curve (AUC) was determined for each protein of interest and normalized by total AUC (total protein) and tubulin AUC [42,43].

### 2.6. Western Blots

The hippocampal proteins (50 µg) obtained from 3 mice treated with either vehicle, melatonin, ketamine, or KET/MEL were separated by electrophoresis in duplicate (SDS-PAGE) on precast 10% polyacrylamide gels (Stain-free 12 BIO-RAD) [41]. Then, the proteins were transferred to PVDF membranes with the BIO-RAD Trans-Blot Turbo system (Bio-Rad, CA, USA) and stained with SYPRO Ruby. Images were obtained using a Chemidoc MP Imaging System (Bio-Rad, Hercules, CA, USA) before blocking. Blots were then incubated with anti-α-CaMKII (1:1000; Abcam, Waltham, MA, USA), followed by a secondary antibody DyLight 680 (Invitrogen, SA5-10042, Thermo Scientific, Waltham, MA, USA). p-CaMKII was stained with an anti-p-CaMKII (Thr 286) (1:1000; Cell Signaling, Danvers, MA, USA) followed by 1:10,000 of secondary antibody DyLight 488 (Invitrogen, SA5-10038, Thermo Scientific, USA). αCaMKII a and p-CaM were detected by ChemiDoc^TM^ MP Imaging System (Bio-Rad, CA, USA). Images were analyzed using the Image Lab software version 6.0 (Bio-Rad Laboratory, CA, USA). The relative amounts of α-CaMKII and p-CaMKII were normalized by the total amount of protein by the maximum and minimum values.

### 2.7. Immunohistochemical Analysis

After the FST, groups of 5 mice/treatment were anesthetized using sodium pentobarbital (50 mg/kg ip) and intracardially perfused with (30 mL) saline solution (0.9% NaCl), followed by a fixative solution (2% paraformaldehyde and 0.02% CaCl_2_) adapted from Gage et al. (2012) [44]. Brains were stored in the fixative solution for 24 h and subsequently preserved in 30% sucrose. Then, they were embedded in OCT (optimal cutting temperature; Thermo Scientific, Waltham, MA, USA).

Coronal slices of 10 µm thickness were obtained using a cryostat (Microm HM 25, Ramsey, MN, USA). The sections were mounted on glass slides and processed as described [32].

Four slices/mouse (2 obtained from the right hippocampus and 2 from the left hippocampus) were incubated overnight at 4 °C with antibodies against p-CaMKII (1:50) developed in a rabbit, followed by a monoclonal anti-α-CaMKII subunit (1:50) developed in a mouse. Secondary antibodies were coupled to DyLight 488 (1:500; source goat) or DyLight 680 (1:500; source donkey Primary antibodies were incubated for 18 h at 4 °C, followed by 1 h incubation at room temperature with secondary antibodies.

Nuclei were stained with DAPI (1 µg/mL). Preparations were mounted in Vectashield and observed using an LS 900 confocal microscope (Carl Zeiss Microscopy, Oberkochen, Germany). The images obtained were analyzed using ZEN Blue software version 3.3 from Zeiss (Jena, Germany). The relative amount of phospho-CaM was normalized by the relative amount of alpha-CaMKII (n = 5). We analyzed four hippocampal slices per mouse.

### 2.8. Determination of CaMKII Activity

CaMKII activity was assessed with the ADP-Glo kinase assay (Promega, V6930, Madison, WI, USA) as described in [20]. Briefly, mice (n = 4) were treated with either the vehicle, ketamine, melatonin, or the combination KET/MEL as described. We obtained homogenates of 4 mice hippocampus per treatment, and total kinase activity was measured with the ADP formation during the enzymatic reaction. Mixtures were incubated for 1 h at room temperature, and chemiluminescence was measured in the Chemi Doc MP imaging system (Bio-Rad). CaMKII activity was assessed by the subtraction of the enzyme activity determined in the presence of 100,000 nM of the CaMKII inhibitor KN-62 [45] from the total kinase activity determined in the homogenates. Determinations were performed in duplicate, and assays were performed three times.

### 2.9. Statistical Analysis

Data that met the criteria for normality (Shapiro–Wilk test) and equal variance were analyzed using One-Way Analysis of Variance (ANOVA), followed by Holm–Sidak’s pairwise multiple comparison test. *p* values ≤ 0.05 data were not met with the normality or equal to the variance criteria were analyzed using the Kruskal–Wallis One Way Analysis of Variance on Ranks, followed by Tukey’s test or Mann–Whitney Sum Test for pairwise multiple comparison.

Graphs were analyzed and plotted using Sigma Plot 12.3 version and GraphPad Prism version 8.0.1.

## 3. Results

### 3.1. Identification of Hippocampal CaMKII by Isoelectric Point and Molecular Weight

**Figure 1:**
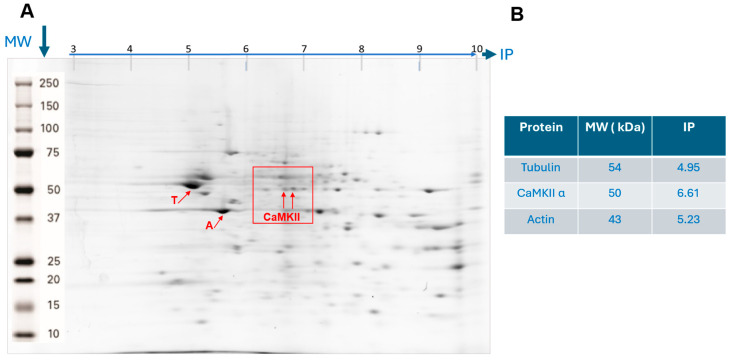
Two-dimensional electrophoretic separation of hippocampal proteins. Total protein of homogenates of hippocampal tissue was separated by 2D-PAGE. (**A**) Shows a representative image of a mouse hippocampal protein pattern stained with SYPRO Ruby. Red arrows indicate the relative mobility of tubulin (T), actin (A), and Ca^2+^/Calmodulin-dependent protein kinase II (CaMKII) separated by isoelectric point (IP) and molecular weight (MW). The specific IP and MW values for these proteins are detailed on the right panel (**B**).

To determine if CaMKII is modified in the hippocampus of mice treated with the KET/MEL combination, we identified CaMKII by isoelectric point (IP) and molecular weight (MW). Thus, we separated proteins of hippocampal (HP) homogenates by two-dimensional electrophoresis (2D-PAGE), and the stained spots were quantified by fluorimetry. Figure 1 shows a representative image of the HP protein pattern. As a reference, we identified tubulin and actin by their IP and MW. We found a single spot of 4.9 IP and 54 kDa that corresponds to tubulin [40] and another single spot of 5.4 IP and 42 kDa that corresponds to actin and an area of spots at 50 KDa and 6.61, 6.64, and 6.66 IP similar to the previously reported for alpha *α*-CaMKII and its phosphorylated isoforms [46].

**Figure 2:**
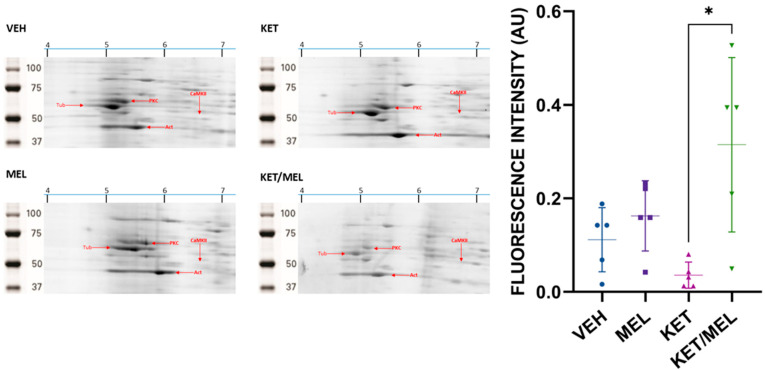
Representative images of α-CaMKII separated by 2D. We quantified the CaMKII in mice treated with either the VEH, MEL, KET, or KET/MEL by fluorimetry. Red arrows indicate: T, tubulin; A, actin; PKC, Protein Kinase C; and α-CaMKII. The graph shows the fluorescence intensity (AU) determined in the 6.61 IP and 50 kDa spot. Data was analyzed using the Kruskal–Wallis One Way Analysis of Variance on Ranks, followed by the Mann–Whitney U Test for pairwise multiple comparisons. * *p* ≤ 0.05.

Once α-CaMKII was identified, we quantified the spot of 6.61 IP and 50 kDa in mice treated with either the vehicle, melatonin, ketamine, or KET/MEL. The amplification of 2D gels, which separated CaMKII derived from mice administered with these treatments, is shown in Figure 2. The graph shows a significant difference in the fluorescence intensity of the *α*-CaMKII spot (H = 10.2, fd = 3, *p* = 0.01), concerning the control group, and the group of mice treated with KET/MEL vs. CTL group (*p* = 0.04), and KET/MEL vs. KET (*p* = 0.005). However, we did not find significant differences in the relative amount of the phosphorylated *α*-CaMKII spots with an IP of 6.64 and 6.6.

### 3.2. The Relative Amount of α-CaMKII in Hippocampus Increases in Mice Treated with KET/MEL Combination

**Figure 3:**
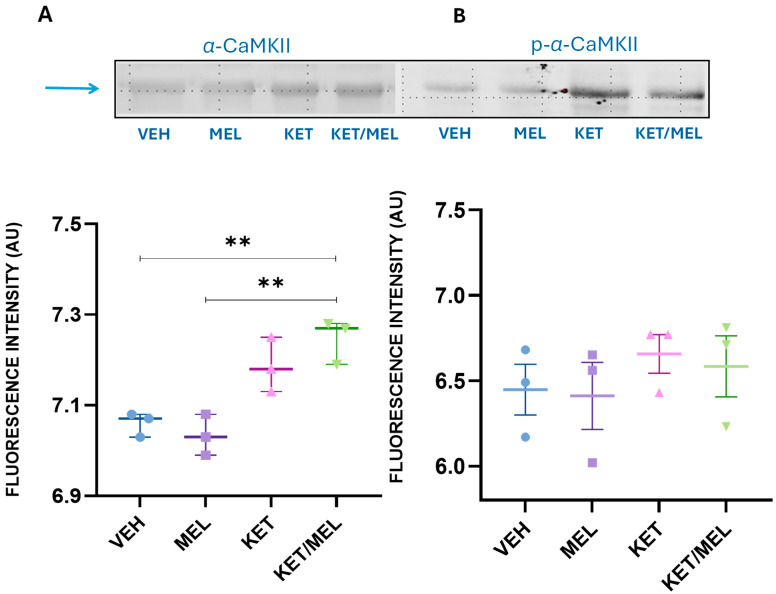
Determination of the relative amount of α-Calcium/Calmodulin Kinase II (α-CaMKII) and phosphorylated α-Calcium/Calmodulin Kinase II (p-α-CaMKII) by Western blot. Mice were administered either vehicle (VEH), melatonin (MEL), ketamine (KET), or the combination of ketamine and melatonin (KET/MEL). Thirty minutes later, the animals were taken to the forced swimming test for 5 min and sacrificed by decapitation immediately afterward. The hippocampus was dissected, and the total homogenate protein was separated by gel electrophoresis. (**A**) shows a representative blot of α-CaMKII identified by a specific anti-α-CaMKII antibody. Data are presented as the mean + SEM of the fluorescence intensity (n = 3). Data were analyzed using One Way Analysis of Variance followed by Holm–Sidak’s Pairwise Multiple Comparison Procedure. Statistical analysis ** *p* ≤ 0.01. (**B**) shows a representative blot of p-α-CaMKII, detected using a specific p-α-CaMKII antibody. The blue arrow indicates the position of a protein with an approximate molecular weight of 50 kDa. The graph shows the fluorescence intensity of the p-α-CaMKII spot for each treatment as an index of relative amount. Data passed the Normality test, but failed the Equal Variance Test, thus, results were analyzed with Kruskal–Wallis One Way of Variance on Ranks.

To corroborate that the relative amount of *α*-CaMKII was augmented after the KET/MEL treatment, we determined the amount of alpha *α*-CaMKII by Western blot (Figure 3).

We found a significant difference between treatments (F_(3,8)_ = 14.11, *p* ≤ 0.001). The group of mice treated with the KET/MEL combination in comparison with the vehicle (*p* = 0.003) and between KET/MEL vs. melatonin (*p* = 0.01), ketamine vs. melatonin (*p* = 0.01), and ketamine vs. vehicle (*p* = 0.03) (Figure 3A). Although we did not find significant differences in the relative amount of phosphorylated CaMKII (Figure 3B; p-*α*-CaMKII), we observed a slight increase in the amount of this protein. Thus, to define if there are changes in the relative amount of p-CaMKII, we measured the amounts of p-CaMKII by immunostaining.

### 3.3. Differential Subcellular Distribution of p-CaMKII Is Observed in Cells of the Dentate Gyrus of Mice Treated with MEL or KET/MEL Combination

**Figure 4:**
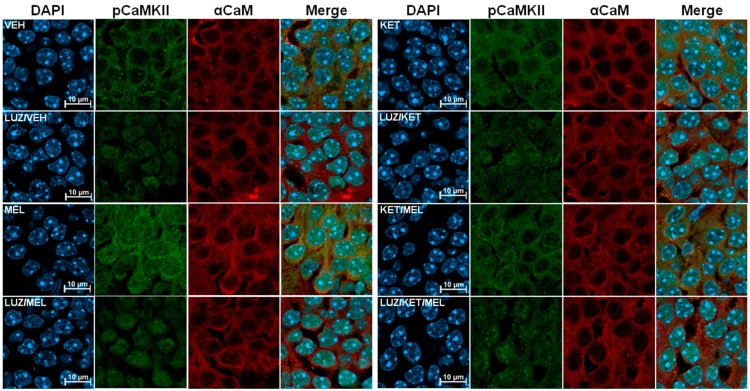
The effect of the KET/MEL combination and Luzindole on the relative levels of phospho-CaMKII in the mouse hippocampus. Representative images of a single optical section of 140 nm acquired from animals administered either vehicle (VEH), melatonin (MEL), ketamine (KET), or the combination of ketamine and melatonin (KET/MEL) as described in the Methods Section. Another group of animals was injected intraperitoneally (i.p.) with 10 mg/kg of Luzindole (LUZ) 15 min before the administration of VEH, MEL, KET, or KET/MEL combination. After 30 min, mice were subjected to the forced swimming test for 5 min, after which they were anesthetized, perfused, and brains fixed. The brains were sectioned in 10 µm slices and immunostained with DAPI to label the nuclei (blue). Additionally, slices were stained with an anti-phospho-CaMKII antibody to detect phosphorylation at threonine 286, followed by DyLight 488 antibody (green). α-CaMKII was labeled using a specific primary antibody and a DyLight 680 secondary antibody (red). Images were acquired using an inverted confocal microscope (LSM900 Carl Zeiss, Oberkochen, Germany). Scale bar = 10 µm.

To define if the activation of CaMKII occurs after acute administration of KET/MEL combination associated with antidepressant-like effects, mice received either VEH, melatonin, ketamine, or KET/MEL combination under an acute administration protocol, and the brains were extracted. Figure 4 shows brain slices that were stained with a specific anti-p-CaMKII antibody (green) and an anti-*α*-CaMKII antibody (red). Also, to identify the nuclear compartment, we stained these slices with DAPI. As shown in Figure 4, in the slices of mice treated with vehicle, the label of p-CaMKII was distributed mainly in the perinuclear area as small green dots. By contrast, in mice treated with melatonin, an increased staining of p-CaMKII was observed in the perinuclear areas and in the nucleus (Figure 4). In contrast, in slices derived from mice treated with ketamine, we observed a diffuse staining around the nuclei and a weak label in the nucleus (Figure 4). Importantly, after KET/MEL combination treatment, we observed scarce labeling with the p-CaMKII antibody in the nuclear compartment and a well-defined perinuclear ring (Figure 4). Remarkably, the distribution and the relative amount of the α-CaMKII (red label) did not change with either of the four treatments.

We also explored whether binding melatonin to melatonin receptors activates the CaMKII signaling pathway. Animals that received either treatment, VEH, melatonin, ketamine, or KET/MEL, were previously treated with luzindole, a non-selective inhibitor of MT_1_ and MT_2_ receptors. We observed an overall decrease in the fluorescence intensity in mice pretreated with luzindole, and the nuclear label of p-CaM diminished in the nucleus of mice treated with luzindole and melatonin (Figure 4). In addition, in the slices derived from mice treated with LUZ and KET/MEL, we observed the CaMKII label as small dots in some nuclei (Figure 4). Interestingly, we observed that in the group pretreated with luzindole plus melatonin, the dots of the p-αCaMKII label were still observed in the nuclei (Figure 4), suggesting that luzindole partially inhibited the p-CaMKII translocation to the nuclei. We did not observe changes in the α-CaMKII label distribution among groups, neither of the four treatment groups, nor the luzindole pretreated groups.

### 3.4. Relative Levels of Phosphorylated CaMKII in the Dentate Gyrus Cells in Mice Treated with the KET/MEL Combination

**Figure 5:**
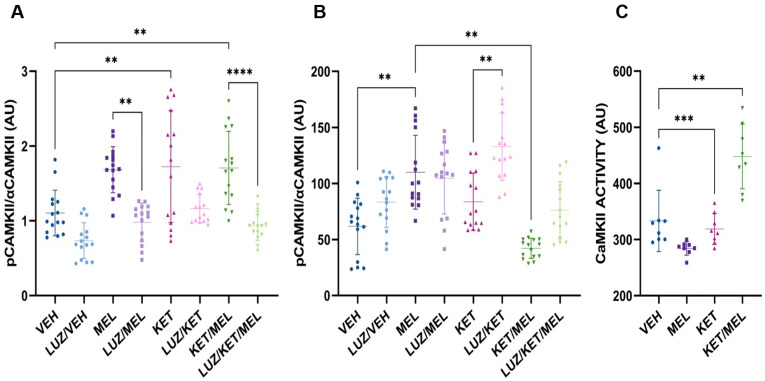
Total and relative nuclear amounts of p-CaMKII and its activity in the hippocampus. Hippocampal slices were obtained from mice administered vehicle (VEH), melatonin (MEL), ketamine (KET), the combination ketamine/melatonin (KET/MEL), or injected i.p. with 10 mg/ of luzindole (LUZ) 15 min prior to treatment. The slices were stained with both anti-p-CaMKII and anti-α-CaMKII antibodies, and fluorescence intensity (arbitrary units of intensity, AUI) was quantified in the same hippocampal region. (**A**) shows the relative amount of p-CaMKII normalized by total α-CaMKII determined across 20 slices (n = 5 mice). (**B**) depicts the relative amount of nuclear p-CaMKII measured in the nuclei of 20 slices (3 slices/mouse). Fluorescence intensities were analyzed using ZEN Blue software (Zeiss). (**C**) illustrates CaMKII activity in the hippocampus, determined using the ADPGLO assay as described in Section 2. The graph represents CaMKII activity measured as the difference between the total luminescence generated by all kinases and the luminescence produced in the presence of KN62, a specific CaMKII inhibitor. Data were analyzed using One-Way ANOVA followed by Tukey’s multiple comparisons tests, and are presented as the mean ± SEM. Significance levels are indicated as follows: ** *p* < 0.001, *** *p* < 0.002, **** *p* < 0.0001.

To study the subcellular distribution of p-CaMKII, we quantified the fluorescence intensity of α-CaMKII and p-CAMKII in the total cell area and in the nuclear compartment (Figure 5A,B). There were significant changes in the relative amount of α-CaMKII determined by immunofluorescence staining in brain slices derived from mice treated with either the vehicle, melatonin, ketamine, or KET/MEL (H = 60.9, fd = 7, *p* ≤ 0.001). As shown in Figure 5A the ratio of p-α-CaMKII α-CaMKII in the group of mice treated with ketamine and KET/MEL increased with respect to the vehicle group (*p* = 0.067 and *p* = 0.002, respectively) (Figure 5A) and significant changes were observed in the p-α-CaMKII/α-CaMKII ratio between each treatment compared to the groups pretreated with luzindole inhibitor plus their respective pharmacological treatment.

In contrast, in the group treated with melatonin, we observed an increase in the relative amount of nuclear p-CaMKII with respect to the vehicle control mice (F_(7112)_ = 15.62, *p* = 0.0024) (Figure 5B). Altogether, these results strongly suggest that p-CaMKII is translocated to the nucleus in cells of the dentate gyrus of mice treated with melatonin, in contrast with the cells of mice treated with KET/MEL, where p-CaMKII remains in the cytoplasm.

Increased levels of the active form of CaMKII are present in the hippocampus of mice treated with KET/MEL.

Therefore, to corroborate that CaMKII was activated by the KET/MEL, we measured the activity of this enzyme in hippocampal extracts by the ADP-Glo method. Using KN62, a specific inhibitor of the α-CaMKII, we determined the activity as the subtraction of the relative amount of total luminescence generated by all the kinases and in the presence of the inhibitor. Figure 5C shows a significant increase in the CaMKII activity, in hippocampal extracts, Kruskal–Wallis’ U test showed significant differences between treatments (H = 23.24, fd = 3, *p* ≤ 0.001), and significant differences between the groups treated with the KET/MEL combination and vehicle-treated group (*p* < 0.0001), and ketamine vs. vehicle-group (*p* = 0.001). Our data indicate that the KET/MEL treatment increases the CaMKII activity in the hippocampus.

## 4. Discussion

**Figure 6:**
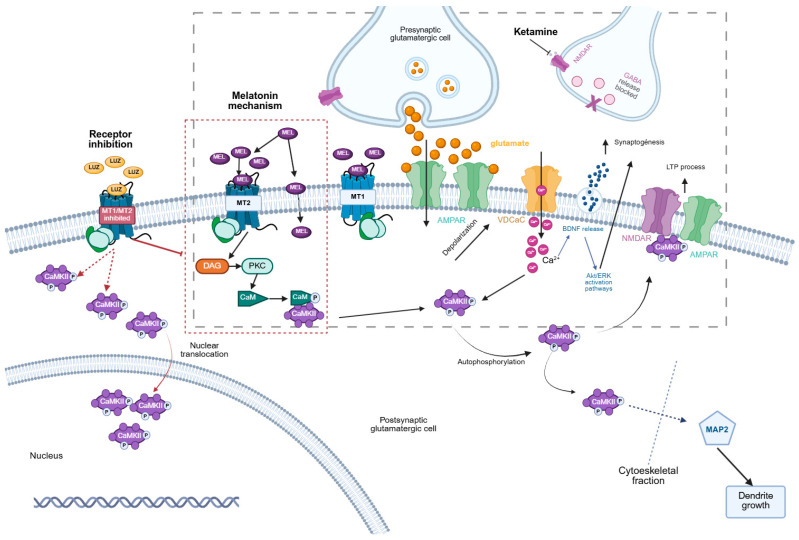
A schematic representation of CaMKII participation in the mechanism underlying the antidepressant-like effects of ketamine/melatonin. Melatonin (MEL) activates MT_1_ and MT_2_ receptors, leading to the production of inositol triphosphate (IP3) and diacylglycerol (DAG). These molecules, in turn, increase intracellular Ca^2+^ concentrations and activate protein kinase C (PKC), respectively. Ca^2+^ binds to calmodulin (CaM), activating it. PKC phosphorylates CaM, enabling its translocation to other subcellular compartments to interact with target proteins. One such target is calmodulin kinase II (CaMKII). CaM binds to CaMKII and activates it, inducing autophosphorylation. Activated CaMKII phosphorylates proteins such as MAPs or associates with actin to enhance dendrite growth and neuroplasticity. Melatonin (MEL) also induces CaMKII translocation to the nucleus, where CaMKII expression is upregulated. On the other hand, ketamine (KET) acts by antagonizing NMDA receptors on GABAergic interneurons, promoting glutamate release. This glutamate release depolarizes the postsynaptic membrane, allowing Ca^2+^ entry and increasing intracellular Ca^2+^ levels, which activate CaM. CaM binds to α-CaMKII, and the active α-CaMKII remains in the cytoplasm, contributing to dendrite growth, neurogenesis, and neuronal connectivity. In the presence of luzindole (LUZ), a non-selective MT1 and MT2 antagonist, nuclear translocation and the increase in the relative amount of CAMKII are blocked. Black continuous arrows depict the common downstream protein activation. The red continuous and dashed arrows indicate protein nuclear translocation. A vertical line head arrow means inhibition of a downstream pathway. A gray dashed rectangle illustrates the general signaling pathway with ketamine in the neuron. And the red dashed rectangle illustrates the specific MT_2_ receptor signaling pathway.

CaMKII is a multimeric enzyme that plays a crucial role in neurodevelopment and neuroplasticity, particularly during neurogenesis and dendrite formation. In previous studies, we demonstrated that this enzyme participates in the formation of new neurons elicited by the KET/MEL combination in cultures of olfactory neuronal precursors. In this paper, we studied whether KET/MEL combination administered ip in doses that cause anti-depressant-like effects to mice that are 250 times and 86 times lower than the LD 50, respectively, according to PubChem Data Base. These doses were able to activate the alpha isoform of CaMKII in the hippocampus. Our results showed that CaMKII is activated after administration of this combination downstream of melatonin receptors and translocated to the nuclei (Figure 6).

Our first approach was to identify the CaMKII isoforms using 2D electrophoresis. The addition of a phosphate group covalently bound to the hydroxy group of serine, threonine, or tyrosine amino acids in proteins results in the addition of negative charges. These charges confer a more basic IP to the proteins, making the phosphorylated isoforms visible [47]. We identified three isoforms of α-CaMKII, and the relative amount of the first isoform (the more acidic one) of α-CaMKII increased in mice treated with the KET/MEL combination [46]. The IP and MW of tubulin and actin, abundant in the brain, were used as a reference to verify the relative mobility of proteins [48]. A previous report indicated that ketamine increases global protein synthesis in the hippocampus of C57BL/6 mice by initially inhibiting eEF2K and CaMKII, which are crucial enzymes for the antidepressant-like effects of ketamine [49]. Therefore, the increased levels of α-CaMKII observed in the hippocampus of mice treated with the KET/MEL combination are consistent with increased protein synthesis reported in C57BL/6 mice treated with an acute dose of ketamine. This finding was corroborated by Western blot, which showed increased amounts of α-CaMKII recognized by a specific antibody.

CaMKII is a holoenzyme activated by the Ca^2+^-CaM complex. When Ca^2+^ binds to CaM, the protein undergoes a conformational change, adopting a bell-shaped structure, which is its activated form. The activated Ca^2+^-CaM can then bind to CaMKII, leading to its auto-phosphorylation at T286 amino acid, thereby making CaMKII independent of further Ca^2+^-CaM activation. Consequently, phosphorylated CaMKII at T286 is considered its active form. Once activated, CaMKII phosphorylates various proteins, which are crucial for the antidepressant effects, among other functions [50,51].

Therefore, in this study, we explored the involvement of the active form of α-CaMKII in the antidepressant effects induced by melatonin, ketamine, or KET/MEL combination by measuring the relative amounts of p-α-CaMKII, phosphorylated at T286 amino acid using Western blots. Although we observed an increase in the total relative amount of α-CaMKII, suggesting its enhanced expression, the difference in the amount of p-α-CaMKII was not significant. Notably, chronic treatment with the antidepressants desipramine and reboxetine up-regulated CaMKII in the soma of neurons [52].

To more precisely quantify the relative amount of active p-α-CaMKII, we used double immunofluorescence labeling of hippocampal slices combined with confocal microscopy. This method allows for the quantitation of specific proteins per cell and the observation of their subcellular distribution. Our data indicated increased relative amounts of p-α-CaMKII in the hippocampus of mice treated with ketamine or the KET/MEL combination. Notably, we observed a differential subcellular distribution of activated p-α-CaMKII and enhanced relative levels of this protein in the nucleus of the dentate gyrus in the hippocampus of mice administered melatonin.

Evidence of changes in the subcellular distribution of CaMKII in neurons of the dentate gyrus of Sprague–Dawley rats has been demonstrated following ketamine administration. The translocation of β-CaMKII to the postsynaptic density protein 95 (PSD95) has been reported and associated with enhanced synaptic plasticity in the dentate gyrus and antidepressant effects [53]. Additionally, the membrane-permeable CaMKII inhibitor tatCN21 blocks the subcellular redistribution of CaMKII in cultured hippocampal neurons. This inhibitor impedes the accumulation of CaMKII at postsynaptic densities and its clustering in dendrites, suggesting that activated CaMKII can be translocated to different subcellular compartments [31]. Moreover, nuclear translocation of CaMKII δ3 in dopaminergic neurons is involved in the increased expression of BDNF. Similarly to α-CaMKII, δ3 CaMKII contains a nuclear localization sequence [54].

In our study, we observed a 78% increase in nuclear translocation of p-α-CaMKII in dentate gyrus neurons of mice treated with melatonin compared to the vehicle-treated group (Figure 6). In addition, ketamine-treated mice showed an increase of 35%. In the mice treated with the KET/MEL combination, we did not observe nuclear translocation of p-α-CaMKII. On the contrary, decreased levels of 32% regarding the vehicle group were observed.

Importantly, nuclear translocation of CaMKII δ3 is prevented when this enzyme is phosphorylated at Ser332, causing it to remain in the cytoplasm. In contrast, dephosphorylation of Ser332 by Protein Phosphatase-1, downstream of Dopamine D2 receptor activation, triggers the nuclear translocation of CaMKII δ3 [55]. Thus, our results suggest that melatonin induces nuclear translocation of p-α-CaMKII, while ketamine causes p-α-CaMKII to remain in the cytoplasm. Notably, p-α-CaMKII appears as cytoplasmic dots around the nuclei in hippocampal neurons of mice treated with melatonin.

Altogether, the evidence suggests that ketamine may induce the phosphorylation of Ser332 amino acid of α-CaMKII, preventing its translocation to the nucleus. Thus, in the hippocampal neurons of mice treated with the KET/MEL combination, the effect of ketamine overrides the melatonin-induced nuclear translocation of p-α-CaMKII, indicating that the KET/MEL combination elicits a differential regulation of the subcellular distribution of α-p-CaMKII. Additionally, it is possible that the differential regulation of CaMKII functions in the cytoplasm and nucleus contributes to an overall antidepressant-like effect that is additive with the KET/MEL treatment [32]. In this regard, CaMKII activity is initially inhibited by autophosphorylation at pT305, followed by activation through autophosphorylation at T286, 20 min after ketamine administration. The initial autoinhibition of CaMKII activity by ketamine concurs with increased protein synthesis, which has been implicated in the antidepressant actions of ketamine [49]. These findings align with the increased α-CaMKII expression observed here after treating mice with KET or the KET/MEL combination. Moreover, we confirmed the increased levels of p-α-CaMKII in mice treated with the KET/MEL combination by measuring its activity through ADP formation in vitro. Remarkably, we found active CaMKII 30 min after KET/MEL administration, corroborating the results obtained from specific labeling of p-α-CaMKII and quantified by confocal microscopy.

Additionally, in this paper, we demonstrated that α-CaMKII is activated by melatonin receptor stimulation. Luzindole, a non-selective competitive melatonin receptor antagonist [56], blocked the increase in the relative amount of p-α-CaMKII elicited by melatonin, ketamine, and the KET/MEL combination, indicating that α-CaMKII activation occurs downstream of these receptors (Figure 6). Moreover, we showed that CaMKII translocation to the nuclei is also prevented by a dose of luzindole previously reported as an effective antagonist [56,57].

It is important to mention that the treatments were administered in the dark phase of the photoperiod at ZT 18. Therefore, at this time, their endogenous melatonin was synthesized by the pineal gland and released to the cerebrospinal fluid [3,57].

Therefore, activated CaMKII is similar in either vehicle, the melatonin or ketamine groups. Hence, luzindole, the MT1/MT2 receptor antagonist, caused the same effect in the vehicle, melatonin, and ketamine groups. Notably, the KET/MEL combination caused a robust increase in the amount of p-α-CaMKII, probably by a summatory effect of endogenous melatonin and exogenously administered ketamine and melatonin.

The differential subcellular distribution of α-CaMKII induced by melatonin, ketamine, and the KET/MEL combination is relevant for the antidepressant effects of these compounds and has physiological implications. For instance, nuclear CaMKII promotes the transcription of proteins through CREB phosphorylation, which enhances transcription and protein synthesis [58,59].

In the cytoplasm, CaMKII plays a critical role in neurite extension, synaptogenesis, and neuronal survival [55]. Remarkably, our results suggest that nuclear translocation of α-CaMKII may increase protein synthesis through CREB phosphorylation, while its presence in the cytoplasm may contribute to increased dendrite formation and neurogenesis in the hippocampus of mice, as previously demonstrated (Figure 6) [17,22,23]. Notably, the subcellular redistribution of CaMKII can direct it to specific compartments to phosphorylate its substrates, promoting neuronal survival and connectivity with other neurons, thereby enhancing neuroplasticity. All these functions play a key role in the treatment of major depression [60].

Previous results published by our group showed that acute administration of KET/MEL combination reduced the immobility time from 59.02 ± 4.4 to 13.5 ± 2.01 (approximately 80%) in the forced swimming test. Furthermore, in the tail suspension test, the combination of KET/MEL produced a similar effect. These results showed that KET/MEL treatment causes a robust antidepressant-like effect in mice [32]. Additionally, stimulation of neurogenesis was already reported in mice administered 1.5/16 mg/kg ketamine/melatonin. Furthermore, we demonstrated that olfactory neuronal precursors incubated with this combination at 10^−7^ M of each compound elicited neurogenesis. This effect was abolished by the CaMKII inhibitor, the compound KN62 [23,32].

In this work, we studied the participation of CaMKII in the signaling pathways of the hippocampus of mice treated with a ketamine/melatonin combination. Remarkably, it has been extensively documented the role of CaMKII in antidepressant-like effects of compounds such as ISSRs, ketamine, and melatonin, and neurogenesis [3,32,61].

## 5. Conclusions

In this paper, we found that the KET/MEL combination and ketamine alone augmented α-CaMKII expression in the hippocampus of mice. Notably, melatonin induces the nuclear translocation of active p-α-CaMKII downstream of melatonin receptors in the neurons of the dentate gyrus. In mice treated with the KET/MEL combination, p-α-CaMKII remains in the cytoplasm. These results suggest the involvement of CaMKII in neurodevelopmental processes and the antidepressant-like effects induced by melatonin and KET/MEL in mice. These findings provide insights into the molecular events related to the KET/MEL combination in the hippocampus. However, further studies are necessary to determine the KET/MEL combination effects on various behavioral paradigms and clinical studies to test the efficacy of the KET/MEL combination in humans.

## Data Availability

The original contributions presented in this study are included in the article. Further inquiries can be directed to the corresponding author.

## Abbreviations

The following abbreviations are used in this manuscript:

α-CaMKII	alpha calmodulin kinase II
AUC	area under curve
AMPA	a-amino-3-hydroxy-5-methyl-4-isoxazolepropionic acid
CaMKII	calcium/calmodulin-dependent protein kinase II
CaM	Calmodulin
CHAPS	3-[(3-Cholamidopropyl) dimethylammonio]-1-propanesulfonate
CREB	cyclic AMP response-binding protein
Fd	freedom degree
HP	Hippocampus
IP	isoelectric point
ip	intraperitoneally
LUZ	luzindole
KET	ketamine
KET/MEL	ketamine/melatonin combination
LTP	long-term potentiation
MEL	melatonin
MW	molecular weight
NMDA	*N*-methyl-D-aspartate
NMDAR	*N*-methyl-D-aspartate receptor
MT_1_	melatonin receptor T_1_
MT_2_	melatonin receptor T_2_
p-CaM	phosphorated calmodulin
p-α-CaM	phosphorated calmodulin
PKC	protein kinase C
Ser332	serine 332
T238	threonine 238
VEH	vehicle
ZT	Zeitgeber time

## References

[1] Ragguett R.-M., Tamura J.K., McIntyre R.S. (2019). Keeping up with the Clinical Advances: Depression. CNS Spectr..

[2] Shokri-Mashhadi N., Darand M., Rouhani M.H., Yahay M., Feltham B.A., Saraf-Bank S. (2023). Effects of Melatonin Supplementation on BDNF Concentrations and Depression: A Systematic Review and Meta-Analysis of Randomized Controlled Trials. Behav. Brain Res..

[3] Estrada-Reyes R., Valdés-Tovar M., Arrieta-Baez D., Dorantes-Barrón A., Quero-Chávez D., Solís-Chagoyán H., Argueta J., Dubocovich M., Benítez-King G. (2018). The Timing of Melatonin Administration Is Crucial for Its Antidepressant-Like Effect in Mice. Int. J. Mol. Sci..

[4] Stein D.J., Picarel-Blanchot F., Kennedy S.H. (2013). Efficacy of the Novel Antidepressant Agomelatine for Anxiety Symptoms in Major Depression. Hum. Psychopharmacol. Clin. Exp..

[5] Venegas C., García J.A., Escames G., Ortiz F., López A., Doerrier C., García-Corzo L., López L.C., Reiter R.J., Acuña-Castroviejo D. (2012). Extrapineal Melatonin: Analysis of Its Subcellular Distribution and Daily Fluctuations. J. Pineal Res..

[6] Yu H., Dickson E.J., Jung S.R., Koh D.S., Hille B. (2016). High Membrane Permeability for Melatonin. J. Gen. Physiol..

[7] Tan D.X., Manchester L.C., Hardeland R., Lopez-Burillo S., Mayo J.C., Sainz R.M., Reiter R.J. (2003). Melatonin: A Hormone, a Tissue Factor, an Autocoid, a Paracoid, and an Antioxidant Vitamin. J. Pineal Res..

[8] Yousefi T., Yousef Memar M., Ahmadi Jazi A., Zand S., Reiter R.J., Amirkhanlou S., Mostafa Mir S. (2024). Molecular Pathways and Biological Roles of Melatonin and Vitamin D Effects on Immune System and Oxidative Stress. Int. Immunopharmacol..

[9] Said A., Shah D., Shah P., Singh B., Anamika F., Aggarwal K., Gupta A., Jain R. (2024). Unlocking the Heart’s Guardian: Exploring Melatonin’s Impact on the Cardiovascular System. Cardiol. Rev..

[10] Sheibani M., Hosseinzadeh A., Fatemi I., Naeini A.J., Mehrzadi S. (2024). Practical Application of Melatonin for Pancreas Disorders: Protective Roles against Inflammation, Malignancy, and Dysfunctions. Pharmacol. Rep..

[11] Slominski A.T., Zmijewski M.A., Semak I., Kim T.K., Janjetovic Z., Slominski R.M., Zmijewski J.W. (2017). Melatonin, Mitochondria, and the Skin. Cell Mol. Life Sci..

[12] Minich D.M., Henning M., Darley C., Fahoum M., Schuler C.B., Frame J. (2022). Is Melatonin the “Next Vitamin D”?: A Review of Emerging Science, Clinical Uses, Safety, and Dietary Supplements. Nutrients.

[13] Miranda-Riestra A., Estrada-Reyes R., Torres-Sanchez E.D., Carreño-García S., Ortiz G.G., Benítez-King G. (2022). Melatonin: A Neurotrophic Factor?. Molecules.

[14] Tancheva L., Lazarova M., Saso L., Kalfin R., Stefanova M., Uzunova D., Atanasov A.G. (2021). Beneficial Effect of Melatonin on Motor and Memory Disturbances in 6-OHDA-Lesioned Rats. J. Mol. Neurosci..

[15] Dubocovich M.L. (1983). Melatonin Is a Potent Modulator of Dopamine Release in the Retina. Nature.

[16] Dubocovich M.L., Delagrange P., Krause D.N., Sugden D., Cardinali D.P., Olcese J. (2010). International Union of Basic and Clinical Pharmacology. LXXV. Nomenclature, Classification, and Pharmacology of G Protein-Coupled Melatonin Receptors. Pharmacol. Rev..

[17] Ramirez-Rodriguez G., Ortíz-López L., Domínguez-Alonso A., Benítez-King G.A., Kempermann G. (2011). Chronic Treatment with Melatonin Stimulates Dendrite Maturation and Complexity in Adult Hippocampal Neurogenesis of Mice. J. Pineal Res..

[18] Liu J., Clough S.J., Dubocovich M.L. (2017). Role of the MT1 and MT2 Melatonin Receptors in Mediating Depressive- and Anxiety-like Behaviors in C3H/HeN Mice. Genes. Brain Behav..

[19] Benítez-King G., Argueta J., Miranda-Riestra A., Muñoz-Delgado J., Estrada-Reyes R. (2024). Interaction of the Melatonin/Ca21-CaM Complex with Calmodulin Kinase II: Physiological Importance. Mol. Pharmacol..

[20] Argueta J., Solís-Chagoyán H., Estrada-Reyes R., Constantino-Jonapa L.A., Oikawa-Sala J., Velázquez-Moctezuma J., Benítez-King G. (2022). Further Evidence of the Melatonin Calmodulin Interaction: Effect on CaMKII Activity. Int. J. Mol. Sci..

[21] Wang L.M., Suthana N.A., Chaudhury D., Weaver D.R., Colwell C.S. (2005). Melatonin Inhibits Hippocampal Long-Term Potentiation. Eur. J. Neurosci..

[22] Domínguez-Alonso A., Valdés-Tovar M., Solís-Chagoyán H., Benítez-King G. (2015). Melatonin Stimulates Dendrite Formation and Complexity in the Hilar Zone of the Rat Hippocampus: Participation of the Ca++/Calmodulin Complex. Int. J. Mol. Sci..

[23] Estrada-Reyes R., Quero-Chávez D.B., Alarcón-Elizalde S., Cercós M.G., Trueta C., Constantino-Jonapa L.A., Oikawa-Sala J., Argueta J., Cruz-Garduño R., Dubocovich M.L. (2022). Antidepressant Low Doses of Ketamine and Melatonin in Combination Produce Additive Neurogenesis in Human Olfactory Neuronal Precursors. Molecules.

[24] Yasuda R., Hayashi Y., Hell J.W. (2022). CaMKII: A Central Molecular Organizer of Synaptic Plasticity, Learning and Memory. Nat. Rev. Neurosci..

[25] Machado-Vieira R., Salvadore G., DiazGranados N., Zarate C.A. (2009). Ketamine and the next Generation of Antidepressants with a Rapid Onset of Action. Pharmacol. Ther..

[26] Duman R.S., Li N., Liu R.J., Duric V., Aghajanian G. (2012). Signaling Pathways Underlying the Rapid Antidepressant Actions of Ketamine. Neuropharmacology.

[27] Li Y.-D., Luo Y.-J., Su W.-K., Ge J., Crowther A., Chen Z.-K., Wang L., Lazarus M., Liu Z.-L., Qu W.-M. (2024). Anterior Cingulate Cortex Projections to the Dorsal Medial Striatum Underlie Insomnia Associated with Chronic Pain. Neuron.

[28] Cooper M.D., Rosenblat J.D., Cha D.S., Lee Y., Kakar R., McIntyre R.S. (2017). Strategies to Mitigate Dissociative and Psychotomimetic Effects of Ketamine in the Treatment of Major Depressive Episodes: A Narrative Review. World J. Biol. Psychiatry.

[29] Bustos F.J., Jury N., Martinez P., Ampuero E., Campos M., Abarzúa S., Jaramillo K., Ibing S., Mardones M.D., Haensgen H. (2017). NMDA Receptor Subunit Composition Controls Dendritogenesis of Hippocampal Neurons through CAMKII, CREB-P, and H3K27ac. J. Cell Physiol..

[30] Weigand A., Horn A., Caballero R., Cooke D., Stern A.P., Taylor S.F., Press D., Pascual-Leone A., Fox M.D. (2018). Prospective Validation That Subgenual Connectivity Predicts Antidepressant Efficacy of Transcranial Magnetic Stimulation Sites. Biol. Psychiatry.

[31] Tao-Cheng J.H., Yang Y., Bayer K.U., Reese T.S., Dosemeci A. (2013). Effects of CaMKII Inhibitor TatCN21 on Activity-Dependent Redistribution of CaMKII in Hippocampal Neurons. Neuroscience.

[32] Estrada-Reyes R., Quero-Chávez D.B., Trueta C., Miranda A., Valdés-Tovar M., Alarcón-Elizalde S., Oikawa-Sala J., Argueta J., Constantino-Jonapa L.A., Muñoz-Estrada J. (2021). Low Doses of Ketamine and Melatonin in Combination Produce Additive Antidepressant-like Effects in Mice. Int. J. Mol. Sci..

[33] Forest A., Swulius M.T., Tse J.K.Y., Bradshaw J.M., Gaertner T., Waxham M.N. (2008). Role of the N- and C-Lobes of Calmodulin in the Activation of Ca^2+^/Calmodulin-Dependent Protein Kinase II. Biochemistry.

[34] Gaertner T.R., Putkey J.A., Waxham M.N. (2004). RC3/Neurogranin and Ca^2+^/Calmodulin-Dependent Protein Kinase II Produce Opposing Effects on the Affinity of Calmodulin for Calcium. J. Biol. Chem..

[35] Hanson P.I., Meyer T., Stryer L., Schulman H. (1994). Dual Role of Calmodulin in Autophosphorylation of Multifunctional Cam Kinase May Underlie Decoding of Calcium Signals. Neuron.

[36] Zalcman G., Federman N., Romano A. (2018). CaMKII Isoforms in Learning and Memory: Localization and Function. Front. Mol. Neurosci..

[37] Chapman P.F., Frenguelli B.G., Smith A., Chen C.-M., Silva A.J. (1995). The α-Ca^2+^/Calmodulin Kinase II: A Bidirectional Modulator of Presynaptic Plasticity. Neuron.

[38] Caffino L., Piva A., Mottarlini F., Di Chio M., Giannotti G., Chiamulera C., Fumagalli F. (2018). Ketamine Self-Administration Elevates ACaMKII Autophosphorylation in Mood and Reward-Related Brain Regions in Rats. Mol. Neurobiol..

[39] Madison D.V., Edson E.B. (1997). Preparation of Hippocampal Brain Slices. Curr. Protoc. Neurosci..

[40] Towbin H., Özbey Ö., Zingel O. (2001). An Immunoblotting Method for High-Resolution Isoelectric Focusing of Protein Isoforms on Immobilized PH Gradients. Electrophoresis.

[41] Laemmli U.K. (1970). Cleavage of Structural Proteins during the Assembly of the Head of Bacteriophage T4. Nature.

[42] Brauner J.M., Groemer T.W., Stroebel A., Grosse-Holz S., Oberstein T., Wiltfang J., Kornhuber J., Maler J.M. (2014). Spot Quantification in Two Dimensional Gel Electrophoresis Image Analysis: Comparison of Different Approaches and Presentation of a Novel Compound Fitting Algorithm. BMC Bioinform..

[43] Natale M., Maresca B., Abrescia P., Bucci E.M. (2011). Image Analysis Workflow for 2-D Electrophoresis Gels Based on ImageJ. Proteom. Insights.

[44] Gage G.J., Kipke D.R., Shain W. (2012). Whole Animal Perfusion Fixation for Rodents. J. Vis. Exp..

[45] Awalsh D., Glass D.B. (1991). Utilization of the Inhibitor Protein of Adenosine Cyclic Monophosphate-Dependent Protein Kinase, and Peptides Derived from It, as Tools to Study Adenosine Cyclic Monophosphate-Mediated Cellular Processes. Methods Enzymol.

[46] Scholz W.K., Baitinger C., Schulman H., Kelly P.T. (1988). Developmental Changes in Ca^2+^/Calmodulin-Dependent Protein Kinase II in Cultures of Hippocampal Pyramidal Neurons and Astrocytes. J. Neurosci..

[47] Benítez-King G., Cazares F., Meza I. (1989). Synthesis and Phosphorylation of Cytoskeletal Proteins during the in Vitro Biogenesis of MDCK Cell Monolayers. J. Cell Sci..

[48] Bryan R.N., Bossinger J., Hayashi M. (1981). Tubulin and Actin Synthesis during Brain Development. Dev. Biol..

[49] Adaikkan C., Taha E., Barrera I., David O., Rosenblum K. (2018). Calcium/Calmodulin-Dependent Protein Kinase II and Eukaryotic Elongation Factor 2 Kinase Pathways Mediate the Antidepressant Action of Ketamine. Biol. Psychiatry.

[50] Tzortzopoulos A., Best S.L., Kalamida D., Török K. (2004). Ca^2+^/Calmodulin-Dependent Activation and Inactivation Mechanisms of AlphaCaMKII and Phospho-Thr286-AlphaCaMKII. Biochemistry.

[51] Hudmon A., Schulman H. (2002). Neuronal Ca^2+^/Calmodulin-Dependent Protein Kinase II: The Role of Structure and Autoregulation in Cellular Function. Annu. Rev. Biochem..

[52] Tiraboschi E., Giambelli R., D’Urso G., Galietta A., Barbon A., de Bartolomeis A., Gennarelli M., Barlati S., Racagni G., Popoli M. (2004). Antidepressants Activate CaMKII in Neuron Cell Body by Thr286 Phosphorylation. Neuroreport.

[53] Abdoulaye I.A., Wu S.S., Chibaatar E., Yu D.F., Le K., Cao X.J., Guo Y.J. (2021). Ketamine Induces Lasting Antidepressant Effects by Modulating the NMDAR/CaMKII-Mediated Synaptic Plasticity of the Hippocampal Dentate Gyrus in Depressive Stroke Model. Neural Plast..

[54] Mishra S., Gray C.B.B., Miyamoto S., Bers D.M., Brown J.H. (2011). Location Matters: Clarifying the Concept of Nuclear and Cytosolic CaMKII Subtypes. Circ. Res..

[55] Shioda N., Sawai M., Ishizuka Y., Shirao T., Fukunaga K. (2015). Nuclear Translocation of Calcium/Calmodulin-Dependent Protein Kinase IIδ3 Promoted by Protein Phosphatase-1 Enhances Brain-Derived Neurotrophic Factor Expression in Dopaminergic Neurons. J. Biol. Chem..

[56] Dubocovich M.L., Mogilnicka E., Areso P.M. (1990). Antidepressant-like Activity of the Melatonin Receptor Antagonist, Luzindole (N-0774), in the Mouse Behavioral Despair Test. Eur. J. Pharmacol..

[57] Ortiz-López L., Pérez-Beltran C., Ramírez-Rodríguez G. (2016). Chronic Administration of a Melatonin Membrane Receptor Antagonist, Luzindole, Affects Hippocampal Neurogenesis without Changes in Hopelessness-like Behavior in Adult Mice. Neuropharmacology.

[58] Sakagami H., Kamata A., Nishimura H., Kasahara J., Owada Y., Takeuchi Y., Watanabe M., Fukunaga K., Kondo H. (2005). Prominent Expression and Activity-Dependent Nuclear Translocation of Ca2+/Calmodulin-Dependent Protein Kinase Idelta in Hippocampal Neurons. Eur. J. Neurosci..

[59] Dahleh M.M.M., Mello C.F., Ferreira J., Rubin M.A., Prigol M., Guerra G.P. (2024). CaMKIIα Mediates Spermidine-Induced Memory Enhancement in Rats: A Potential Involvement of PKA/CREB Pathway. Pharmacol. Biochem. Behav..

[60] Tsui J., Inagaki M., Schulmann H. (2005). Calcium/Calmodulin-Dependent Protein Kinase II (CaMKII) Localization Acts in Concert with Substrate Targeting to Create Spatial Restriction for Phosphorylation. J. Biol. Chem..

[61] Valdés-Tovar M., Estrada-Reyes R., Solís-Chagoyán H., Argueta J., Dorantes-Barrón A.M., Quero-Chávez D., Cruz-Garduño R., Cercós M.G., Trueta C., Oikawa-Sala J. (2018). Circadian Modulation of Neuroplasticity by Melatonin: A Target in the Treatment of Depression. Br. J. Pharmacol..

